# The forensic educational outreach initiative – Bridging the gap between education and workplace

**DOI:** 10.1016/j.fsisyn.2023.100448

**Published:** 2023-12-17

**Authors:** Ray Wickenheiser, Amanda Cadau, Claire Muro, Samantha Whitfield, Carrie McGinnis, Lola Murray, Melissa France, Lyn Niles, Donna Barron, Lori Valentin

**Affiliations:** a New York State Police Crime Laboratory System, Albany, NY, USA; b Hudson Valley Community College, Troy, NY, USA; c The College of Saint Rose, Albany, NY, USA

**Keywords:** Forensic science, Forensic incubator, Educational outreach, Internship, Students, Training

## Abstract

Skills and knowledge gaps frequently exist between forensic educational programs and practical forensic laboratory needs. An educational outreach project involving three post-secondary academic institutions and a large multidisciplinary forensic laboratory was created to provide lectures to students, enable mentorship with forensic scientists, and provide an interactive experience within the forensic laboratory. Mentorship mixer exercises encouraged meaningful interactions between students and scientists, creating opportunities for practical discussion on employment requirements, optimal class selections based on students’ interests and forensic science requirements, and better understanding of the daily tasks and duties of operational forensic scientists. Feedback from students, professors, and forensic mentors have resulted in program improvements which will inform the educational outreach initiative going forward, including broader community outreach.

## 1 Introduction

Creating and maintaining talented labor is a priority for any organization, and forensic laboratories are no exception. One of the four goals in the National Institute of Justice's (NIJ) Strategic Research Plan is to “cultivate a diverse and highly skilled workforce” [1]. This plan acknowledges the importance of workforce development to support successful forensic work. There is a clear need to grow our personnel and attract new hires. Demand for forensic scientists is expected to increase “much faster than average” over the coming years [2]. Laboratories need to be prepared to meet increased requests for forensic services or risk backlogs and other operational issues.

As current practitioners are aware, forensic science is a demanding occupation. The pressures for many start early with challenging coursework in college, continues in the application process with lengthy background investigations, and persists through their career. The behind-the-scenes demands while working in the field include testifying in a court of law, performing proficiency tests, adhering to strict accreditation and quality assurance requirements, involvement in distressing casework, the knowledge that analysis will impact an individual's life, and possibly freedom, and expectations to continue training and improvement. This entire time, ethical standards must be continuously maintained.

In recent years, the New York State Police Crime Laboratory System (NYSP CLS) has experienced a decline in the number of applicants for forensic positions. This has been seen across the country with a 4% decrease in law enforcement employment, including both sworn officers and non-sworn employees, between March 2020 and August 2022 [3]. An obvious factor is the inability for many government laboratories to offer salaries that can compete with private industry. Additionally, many forensic positions require candidates to pass a comprehensive background investigation. The applicants that do make it through the application process are a large investment– a year of training can cost $100,000 or more. Thus, retention of scientists is vital to the success of an organization. Since careers in forensics are still appealing, laboratories can employ strategic initiatives to recruit and maintain top applicants.

Recruitment of potential applicants starts with engaging high school [4] and college students so that they can learn more about a career in forensics early in their academic careers. There are many benefits to students participating in these types of recruitment activities such as seeing what an average workday looks like and learning the specific educational requirements for a discipline they might be interested in. This experience is valuable because, for many students, their sole impression of forensic science is based on inaccurate entertainment media depictions. As these representations are rarely authoritative, definitive, or correct, it is helpful for students to see what a real career in forensics is like before they make a commitment, and a forensic laboratory makes an investment.

Partnering with higher education institutions is mutually beneficial to both the laboratories and the academic institutions [5]. While many forensic programs aim to prepare students for a career in the field, some still do not adequately cover specific aspects of the job, including required coursework [6]. By partnering with a college or university, a laboratory can inform them of the coursework they view as most needed in new hires. Enrolling in a Forensic Science Education Programs Accreditation Commission (FEPAC) accredited school or program ensures that a program includes necessary forensic science educational requirements and recognition for adherence to those standards [7]. However, even FEPAC accredited programs do not address the gap that still exists between undergraduate curriculum and the reality of working in a forensic lab [8]. There has been previous discussion about rethinking forensic coursework to focus on this issue [[9], [10], [11], [12]], and here we provide an additional approach.

For students interested in undergraduate or graduate research, laboratory staff can develop programs such as an internship to aid in laboratory activities, like validation, database building, or research work. This relationship is symbiotic as the students gain experience working on real-world projects while the laboratory gains man-hours to help complete them while also enriching the applicant pool.

Besides recruiting applicants, it is also important to develop the skills of existing laboratory employees. Presenting information to students and faculty provides scientists with public speaking experience, which is critical in a profession that requires regular courtroom testimony. Mentoring college students allows employees to hone mentoring and leadership skills, in addition to the benefits of mentoring for the students [13]. Lastly, working with universities to develop and validate methods offers opportunities to engage in project design and execution. All of these elements combine to create a program that provides real value to the forensic community but can also increase employee job satisfaction by diversifying job duties and allowing a structure for outreach and enrichment.

## 2 Educational outreach and incubator concept proposal

### 2.1 Goals

The partnership between the NYSP CLS and the academic institutions aims to increase the quality and quantity of applicants for laboratory employment. Various methods have been explored to attempt to bridge the gap between the forensic laboratory and post-secondary science educational programs.

Leading laboratories should be innovating and researching in addition to maintaining a quality caseload output. However, finding resources with which to research and innovate when personnel are busy with a demanding caseload is a constant challenge. This partnership targets broad-reaching benefits for:

1) Students, by enhancing the students' technical skills by providing direct access to laboratory techniques.
2) Forensic laboratories, by providing more resources.
3) Academic Programs, by refining class topics and content towards those applicable to and in use by forensic laboratories.
4) Academic Research, by improving focus of research projects through exposure to real-world applications in forensic science.
5) Scientific community as a whole, through meaningful collaborations that lead to impactful publications and presentations.

## 3 Methods

The NYSP CLS developed the Forensic Investigative Curriculum (FIC) School educational outreach initiative to provide students with more information about forensic science careers. The program is designed to have scientists, students, and professors meet several times per college semester, with each meeting focused on a different discipline within forensic science. Local colleges and universities host FIC School events at their facilities, allowing students and educators from multiple institutions to gather. After an initial general introductory session about the CLS and information pertaining to pursuing a career in forensic science, subsequent presentations provide more detailed information about the various laboratory disciplines represented in the CLS. These presentations include Biological Sciences (DNA), Seized Drugs (Drug Chemistry), Toxicology, Firearms, and the Forensic Identification Unit (Latent Prints). Presentations were given by the scientists and professionals that work within each forensic discipline, and provide an overview of the discipline, what a ‘day in the life’ of a scientist might look like working in the laboratory, and information for students about any specific degree, coursework, or certifications that are recommended or required. After each lecture session, students were provided the opportunity to meet with forensic scientist mentors representing the various disciplines during the Mentor Mixer informal meet and greet session. These sessions also empowered students to make informed decisions regarding their own academic and professional paths while also networking with other students and faculty from other institutions.

During the final session of the semester, the FIC School program culminates with a presentation about Quality Assurance in the laboratory and a tour of the Forensic Investigation Center, which is offered to students by invitation only. To be invited to tour the laboratory, students must have attended at least two of the informational sessions. During the tour, students and educators are guided through the laboratory and are provided demonstrations of various tools and techniques used by some of the sections, given the opportunity to see typical casework evidence items, and interact with the scientist performing their duties.

With an hour of professional development workshop in each month of the semester for the mentors, professional forensic scientist students gain experience with Mentorship, Leadership, Communication, Presentation Skills, Team Dynamics and Effective Meetings. This training enriches their professional skillset with tools to effectively mentor students and apply to career development and even promotional opportunities. Most professional development content offered to forensic scientists is technical in nature and honing in on soft or “essential” skills sets the professional development in this program apart. By developing better mentors, the project develops more effective ways to deliver valuable information to the students and to better find the combination of interest and aptitude to guide their career path. The monthly time expectations of scientist mentors is the 1.5-h session with the students, the 1-h leadership program, and up to an additional 1 h to prepare presentations, tour demonstrations, or work on planning other aspects of the program. There is minimal impact to the caseload of each forensic scientist.

Virtual mentor sessions were explored during the first semester of the pilot program but were not as successful as expected. Students were offered opportunities to sign up for one-on-one or small group sessions to speak directly (via a virtual platform) with a forensic science professional to answer any questions they had about their degree path or a career in a specific forensic discipline. Participation in the virtual mentoring was minimal, attributed to the FIC School program being so new; however, this approach is something that may be explored again for inclusion in future FIC School programs.

To promote the FIC School program, a brochure was created to describe the Crime Laboratory System and sections of the laboratory and introduce the educational outreach program. Additionally, a flyer was created to outline important FIC School event information, such as session dates and locations. The flyer also includes a unique QR code linking users to the FIC School's own web page. The brochure and flyer are used for distribution to our current educational partners and also to other colleges, universities, and even high school Science, Technology, Engineering, and Math (STEM) programs to promote future educational partnerships.

The FIC School web page (https://sites.google.com/view/ficschool) was created to keep interested students and educators up to date with the program's scheduled events and provide links to relevant forensic-related publications and resources, as well as current job offerings at the New York State Police. The website provides hyperlinks to the FIC School program's intake form for interested students to enroll in the program, a survey for students to submit following completion of the program, current employment postings within the laboratory, and provides brief introductions to each of the forensic science professionals involved in the pilot program. Local news media outlets and school media departments that featured the FIC School during the various presentation sessions are also displayed on the web page for future students to view.

Each laboratory section represented in the program was responsible for creating and presenting a brief but detailed 15–20-min lecture about their discipline. Content covered the basics of the discipline, such as what the forensic scientists in that discipline were responsible for analyzing, the basic overview of analytical test methods, equipment used in analysis, and general educational requirements to hold the position of forensic scientist or laboratory technician. After the lecture portion of the session, students were able to speak with the mentors in a one-on-one (or realistically two or three-on-one) informal setting. NYSP CLS management met with educators to align educational programs with forensic discipline requirements and seek projects for collaboration. Students used questions provided by the program as a starting point for discussion, however, those discussions quickly morphed into detailed dialogue about the mentors’ experiences in entering the field of forensic science. Mentors detailed their educational journey as well as their professional timeline in the laboratory. The students overwhelmingly had the same general question – “What does a day in the life of a forensic scientist look like?” This became the basis for the pilot year program.

Forensic professionals also participated in a forensic leadership development program delivered by The College of Saint Rose, one of the academic partners. The leadership program was a pilot program that spanned eight months and was designed by an employee at The College who was previously employed as a forensic scientist by the NYSP CLS. The program tested four different learning modalities during the first semester:

1) In-person.
2) Hybrid.
3) Virtual.
4) Asynchronous.

Learning topics were selected by NYSP CLS management:

1) Mentorship/coaching (in-person),
2) Communication (virtual and asynchronous), and
3) Presentations/public speaking (in-person and asynchronous).

Each topic included a total of 60 min of learning content.

## 4 Results and discussion

The Forensic Outreach concept has the potential to leverage the NYSP CLS scientific expertise and infrastructure with educational institutions and, eventually, with private industry to create a unique partnership to develop new analytical methods and technologies while enhancing educational opportunities. The Educational Outreach Initiative began by canvassing laboratory staff who had volunteered for a mentor role, and they advised that dispelling common misconceptions of the field should be a focus of the program. Many students understood that there was a disconnect between the Hollywood version of forensic science and the real-life version. However, they didn't know quite where to draw the line of distinction between reality and television. Mentors tailored their lecture sessions to outline the basic concepts of the discipline as well as illustrate job duties that occur daily.

A total of 18 students participated in the fall 2022 semester of the FIC School, and 16 in the spring semester. Evaluation of the Educational Outreach Initiative was conducted through feedback from the students, the academic institutions, media news coverage, and laboratory personnel. Students completed an anonymous electronic survey evaluating the components of the outreach initiative that they participated in. Results of the electronic survey are listed in Table 1.

**Table 1:** Student electronic survey and participant feedback (n = 8).

Portions of the FIC School Program Participated In?	
Live Presentations	100%
Mentor Mixers (Q&A after presentations)	100%
Virtual Mentoring Sessions	100%
Laboratory Tour/Demonstrations	100%
Rating of the Live (Section-Specific) Presentations	
Excellent	100%
Neutral	0%
Boring	0%
Rating of the Mentor Mixers (Q&A after presentations)	
Excellent	91.3%
Neutral	8.7%
Boring	0%
Rating of the Virtual Mentoring Sessions	
Excellent	81.8%
Neutral	18.2%
Boring	0%
Rating of the Lab Tour and Demonstrations	
Excellent	100%
Neutral	0%
Boring	0%

All the survey respondents rated the presentations by the scientific professionals as excellent. Of the students involved in the outreach initiative, over 91% rated the Mentor Mixers as excellent, further indicating the mentor mixers as a valuable opportunity for them. The opportunity for one-on-one conversations with the forensic professionals provided students with a platform to ask their own questions and learn exactly from the experts what their duties include. Additional feedback from the mentor mixers included how valuable the interaction and speaking with the laboratory staff was, learning more about the forensic industry, and hearing what the laboratory staff enjoy most about their jobs as well as the challenges they face. These components of the program were identified as very beneficial by the students.

Students found the virtual meetings to be beneficial due to the targeted information they received from the laboratory staff during the interaction in a more private and personal setting. Virtual sessions were found to benefit those students who were shy and not as comfortable with the in-person group setting.

The most successful session of the FIC School was the interactive laboratory tour and section demonstrations. The students were separated into three different groups consisting of seven to eight students per group. In the fall 2022 session, the mentors from the seized drugs section of the laboratory demonstrated analysis of a mock case in real-time with the students. This interaction allowed the students a glimpse of what the daily activities of a forensic scientist in seized drugs looks like and get a better idea if the seized drugs discipline would be a career of interest for them. In the spring 2023 session, the bioscience casework section held a detailed demonstration for the students. Again, emphasis was placed on demonstrating the various aspects of a ‘day in the life’ of a forensic scientist assigned to bioscience. Unanimously, the students rated the laboratory tour and demonstrations as excellent.

Individual evaluations of the participating students were also positive and showed growth among many of the students. One student, currently in his second year at Hudson Valley Community College (HVCC) majoring in Chemistry, was a very shy and reserved individual at the beginning of the mentor program. At each event, he grew more comfortable in approaching the mentors and participating in the Q&A sessions. He was also an active participant in the virtual mentor sessions. By the end of the third session, this student participated in two interviews with the local television news stations. Several other students showed growth, too, becoming more comfortable talking to the forensic laboratory professionals. The mentorship program provided sufficient information to allow students to decide if the field of forensics is the field of science they want to enter and make their career.

The laboratory tours and demonstrations were valuable for the students, providing them with applied knowledge and understanding of each section and which discipline they may be interested in joining. Local news media stations attended the laboratory tour and demonstration event to promote the partnership between local colleges and the NYSP CLS and educate the community. News reporters interviewed both students and mentors about the benefits of the partnership. The feedback and interviews were positive and, in return, enhanced the undergraduate forensic programs at each of the academic institutions.

The program has created a positive relationship between the forensic laboratory and the professors at the academic institutions who partnered with the CLS to host the Educational Outreach Program. The professors value the real-life insight into the crime laboratory environment and the insider advice that the mentors provide to the students. Professors with scientific expertise in forensic science related topics have the additional benefit of developing research initiatives in partnership with the forensic laboratory. Educational institutions that participate in the program establish connections with the laboratory mentors to offer targeted advice to the students to help them succeed in obtaining jobs in the forensic field, which is often highly competitive. In the end, both the laboratory and the schools benefit. The program connects students with mentors but also gives mentors the opportunity to evaluate potential candidates for employment and hone professional skills, such as presenting and coaching/mentoring. Increased exposure to forensic scientists, administrators, and their experience assists educators in building their programs. The mentors can help guide and shape the students as they work to complete their education, suggest relevant coursework to take in addition to their regular graduation requirements, and also advise the students on possible internship projects and opportunities. Discussions regarding future program options and improvements include expanding the potential collegiate partnerships and opening the program up local accelerated high school forensic science and STEM students.

Mentors learned how to better communicate information to the younger demographic as well as strengthen their public speaking skills overall. This is a benefit for both seasoned forensic professionals and new hires. Participating in the Educational Outreach Program was rewarding and energizing for the mentors. Oftentimes, it is easy to become entrenched in the daily routine. However, taking time to interact and connect with the students and professors was a unique opportunity for the mentors to remember why they entered the field of forensic science in the first place. The students and professors were very enthusiastic in their participation in the program. The mentors felt appreciated and fulfilled as they were able to share their passion and knowledge. Many of the mentors strived to be the ideal mentor – as they wished had existed when they were in school.

The entire project has been very well received by the public. The Educational Outreach team has been featured in the school news at all the current partner institutions - College of Saint Rose, State University of New York (SUNY) at Albany, and Hudson Valley Community College. Local news channels also visited during the two laboratory tour days. All of this exposure will increase awareness of the program and a better understanding of the field. Ideally, the program can reach more students who may not know much about forensic science and entice them to embark on this morally rewarding scientific career.

The electronic survey also asked the students to identify areas of improvement for the program. Students expressed their desire for additional information and demonstrations about fingerprinting and other sections within the laboratory, in addition to the chemistry and biology sections. Additional feedback for improvement included a request for longer mentor mixer Q&A sessions, more in-depth presentations explaining the daily duties of the laboratory staff, as well as longer live demonstrations.

Further recommendations from students included adding additional laboratory mentors to the program. Some students would like to have interactive time in the laboratory shadowing laboratory staff throughout their workday in addition to internship opportunities. However, crime laboratories are generally understaffed and operating with a casework backlog of some extent. Taking time away from casework is a “hard sell” especially when there is no immediate payoff to the laboratory. The second hurdle is the extensive background check required to allow the intern into the section/laboratory unescorted. For the NYSP, that background check is costly, lengthy, and sometimes difficult to pass for prospective employees let alone student interns. Often, the length of the background check process will exceed the length of the internship itself. This is not ideal for many students who do not want to or cannot wait that long to begin an unpaid internship. Lastly, there is limited or no funding to support internships. Instrumentation, reference materials, and equipment are costly. To divert any of these items from casework is problematic. However, partnering with colleges and universities can help to solve many of these problems. A proposal is being developed to enter a remote internship in partnership with the various schools that have worked with the NYSP CLS in the Educational Outreach Program.

In the proposal, the school will provide the equipment and instrumentation onsite and the professor can supervise the students in their research. The research topic(s) will be of interest to the laboratory - students can focus on an area of research or validation that the laboratory has a need for but lacks the time and/or additional personnel to pursue. Students could come onsite to the laboratory to run final testing once the validation has been planned and tested at the school. One limitation of this concept is that schools may not have the exact same instrumentation as the NYSP CLS or have the correct license for controlled and/or dangerous substances at their institution. Short term in-laboratory work can be supervised without detriment to the forensic laboratory section. This also negates the need for a lengthy background check process. In the above example, the forensic laboratory has minimal resources invested into the internship but can then use the research project to design an abbreviated validation study that can then be used to generate and implement a procedure for the specific type of analysis.

The Educational Outreach team has also been able to brainstorm ideas to assist with employee recruitment and retention that are valuable to the Human Resources department. Working with Human Resources, the team hopes to reduce as many barriers as possible to the employment process. As mentioned previously, the existing background process is extensive and exigent. Often, there is little to no communication to the applicant once the process begins. With team input, laboratory leadership has suggested a more open dialogue with candidates to provide updates throughout the hiring process. Also suggested is a portal or other mechanism of information sharing where the candidate can see the steps needed to gain employment, where they are in the process, and what remains. Both initiatives have been well received and are now being met by an informational fact sheet both read and provided to prospective employees. The website/portal is a larger project that will take more time but can be augmented in the interim by individual outreach to candidates and greater contact via email.

It has also been documented that many candidates are lost during the hiring process. The team has also suggested that Human Resources deploy an anonymous survey to find out why candidates terminate the process prematurely so the organization can work to fix any shortcomings in the hiring process. An additional proposal for the creation of a mailing list where prospective employees can sign up to be notified of new job postings has been accepted and will be implemented. While those technological advancements are in discussion and development, the team has been able to add the mailing list to the Educational Outreach web page to bridge the gap.

The entire Educational Outreach Program has been tailored to benefit the students. The NYSP is committed to the recruitment and retention of valuable, dedicated employees. The goal of the Educational Outreach Program is to interact with driven and motivated STEM students early on in their academic career and guide them into the forensic science career path. The program goals for future improvement are to interact with the students as early on as possible – the team has decided to expand the second year of the program to include select high school students. Senior undergraduate students and graduate students will be given priority to work with the laboratory on collaborative projects that can act as relevant experience for the students as they apply for forensic jobs. The Educational Outreach team is building lasting relationships with the students who will be able to contact their mentors for guidance as they look for employment and beyond as they eventually become colleagues in the same field. The forensic laboratory also benefits from having extended contact with a student to better evaluate a student's potential for long term employment, as well as provide further instruction and one-on-one mentorship within the laboratory setting.

The purpose of the NYSP strategic plan is “to attract and retain diverse and highly skilled talent, develop leaders, and maintain public trust and instill confidence in the agency”. Furthering the goal of the strategic plan, the Educational Outreach team proposed that the laboratory staff an exhibit at the 2023 Great New York State Fair. While staffing for this event was made available to all NYSP laboratory employees, the main contingents are also mentors through the Educational Outreach Program. This event will increase public knowledge of the laboratory, clarify some of the mysticism surrounding forensics, and hopefully encourage more quality students to pursue a forensic science career path.

## 5 Conclusions

The novel Educational Outreach Initiative and FIC School program created several avenues of outreach and growth to NYSP CLS staff and aspiring forensic scientists. The FIC School supported outreach and recruitment efforts of appropriately qualified candidates. Accreditation requirements are very specific regarding the academic requirements to qualify as a forensic scientist. It is ultimately the academic institutions’ responsibility to be aware of these requirements and to ensure that their curriculum includes the required coursework and laboratory components. As such, it is a major advantage to an academic institution to be able to participate in programs such as the FIC School program designed by the NYSP CLS forensic scientists.

Most forensic laboratories operate within a government agency and must adhere to strict practices that dictate local, state, and federal recruitment and hiring. Recruiting and retaining top talent is challenging in an ever-evolving competitive market and it takes an enormous quantity of resources to achieve.

Active recruitment implemented through the novel FIC School program addresses these challenges by:

1) Working with academic institutions to encourage that their curriculum encompasses accreditation requirements and standards, including the FBI QAS for DNA testing laboratories.
2) Exposing students (and potential candidates for employment) to crime laboratory work, offering real-world insights.
3) Early and repeated exposure to the forensic laboratory fosters a collaborative relationship between the laboratory and the academic community, laying the groundwork for a recruitment pipeline.

Direct engagement between forensic scientists and science students provides a unique vantage point for the students to understand more of the nuances of the forensic profession. Students are exposed to the day-to-day realities of working in a forensic laboratory, including quality control, accreditation, evidence handling, triage of evidence, report writing and reviews, and courtroom testimony. Laboratory work within a paramilitary organization requires an appropriate combination of personality and skillset to maintain high quality work in such a regimented environment. Students can learn these important nuances early on and gauge whether it is the right fit for them. A better understanding of forensic laboratory work amongst the student population also strengthens the applicant pool when forensic positions open. The result is a better fit between the applicant and forensic employer and better quality and quantity of applicants, guided by early involvement through educational outreach.

## CRediT authorship contribution statement

**Ray Wickenheiser:** Conceptualization, Methodology, Project administration, Supervision, Visualization, Writing – original draft, Writing – review & editing. **Amanda Cadau:** Writing – original draft, Writing – review & editing. **Claire Muro:** Writing – original draft, Writing – review & editing. **Samantha Whitfield:** Writing – original draft. **Carrie McGinnis:** Writing – original draft, Writing – review & editing. **Lola Murray:** Writing – original draft. **Melissa France:** Writing – original draft. **Lyn Niles:** Writing – original draft. **Donna Barron:** Writing – original draft. **Lori Valentin:** Conceptualization, Writing – original draft.

## Declaration of competing interest

The authors declare that they have no known competing financial interests or personal relationships that could have appeared to influence the work reported in this paper.
